# Antigenicity and Immunogenicity of Differentially Glycosylated Hepatitis C Virus E2 Envelope Proteins Expressed in Mammalian and Insect Cells

**DOI:** 10.1128/JVI.01403-18

**Published:** 2019-03-21

**Authors:** Richard A. Urbanowicz, Ruixue Wang, John E. Schiel, Zhen-yong Keck, Melissa C. Kerzic, Patrick Lau, Sneha Rangarajan, Kyle J. Garagusi, Lei Tan, Johnathan D. Guest, Jonathan K. Ball, Brian G. Pierce, Roy A. Mariuzza, Steven K. H. Foung, Thomas R. Fuerst

**Affiliations:** a School of Life Sciences, The University of Nottingham, Nottingham, United Kingdom; b NIHR Nottingham Biomedical Research Centre, Nottingham University Hospitals NHS Trust and The University of Nottingham, Nottingham, United Kingdom; c W. M. Keck Laboratory for Structural Biology, University of Maryland Institute for Bioscience and Biotechnology Research, Rockville, Maryland, USA; d Department of Cell Biology and Molecular Genetics, University of Maryland, College Park, Maryland, USA; e University of Maryland Institute for Bioscience and Biotechnology Research, National Institute of Standards and Technology, Rockville, Maryland, USA; f Department of Pathology, Stanford University School of Medicine, Stanford, California, USA; University of Southern California

**Keywords:** envelope glycoproteins, HCV, hepatitis C virus, immunogenicity, insect cells, mammalian cells, neutralization, vaccine

## Abstract

The development of a prophylactic vaccine for hepatitis C virus (HCV) remains a global health challenge. Cumulative evidence supports the importance of antibodies targeting the HCV E2 envelope glycoprotein to facilitate viral clearance. However, a significant challenge for a B cell-based vaccine is focusing the immune response on conserved E2 epitopes capable of eliciting neutralizing antibodies not associated with viral escape. We hypothesized that glycosylation might influence the antigenicity and immunogenicity of E2. Accordingly, we performed head-to-head molecular, antigenic, and immunogenic comparisons of soluble E2 (sE2) produced in (i) mammalian (HEK293) cells, which confer mostly complex- and high-mannose-type glycans; and (ii) insect (Sf9) cells, which impart mainly paucimannose-type glycans. Mass spectrometry demonstrated that all 11 predicted *N*-glycosylation sites were utilized in both HEK293- and Sf9-derived sE2, but that *N*-glycans in insect sE2 were on average smaller and less complex. Both proteins bound CD81 and were recognized by conformation-dependent antibodies. Mouse immunogenicity studies revealed that similar polyclonal antibody responses were generated against antigenic domains A to E of E2. Although neutralizing antibody titers showed that Sf9-derived sE2 induced moderately stronger responses than did HEK293-derived sE2 against the homologous HCV H77c isolate, the two proteins elicited comparable neutralization titers against heterologous isolates. Given that global alteration of HCV E2 glycosylation by expression in different hosts did not appreciably affect antigenicity or overall immunogenicity, a more productive approach to increasing the antibody response to neutralizing epitopes may be complete deletion, rather than just modification, of specific *N*-glycans proximal to these epitopes.

**IMPORTANCE** The development of a vaccine for hepatitis C virus (HCV) remains a global health challenge. A major challenge for vaccine development is focusing the immune response on conserved regions of the HCV envelope protein, E2, capable of eliciting neutralizing antibodies. Modification of E2 by glycosylation might influence the immunogenicity of E2. Accordingly, we performed molecular and immunogenic comparisons of E2 produced in mammalian and insect cells. Mass spectrometry demonstrated that the predicted glycosylation sites were utilized in both mammalian and insect cell E2, although the glycan types in insect cell E2 were smaller and less complex. Mouse immunogenicity studies revealed similar polyclonal antibody responses. However, insect cell E2 induced stronger neutralizing antibody responses against the homologous isolate used in the vaccine, albeit the two proteins elicited comparable neutralization titers against heterologous isolates. A more productive approach for vaccine development may be complete deletion of specific glycans in the E2 protein.

## Abstract (precis)

The development of a vaccine for hepatitis C virus (HCV) remains a global health challenge. A major challenge for vaccine development is focusing the immune response on conserved regions of the HCV envelope protein, E2, capable of eliciting neutralizing antibodies. Modification of E2 by glycosylation might influence the immunogenicity of E2. Accordingly, we performed molecular and immunogenic comparisons of E2 produced in mammalian and insect cells. Mass spectrometry demonstrated that the predicted glycosylation sites were utilized in both mammalian and insect cell E2, although the glycan types in insect cell E2 were smaller and less complex. Mouse immunogenicity studies revealed similar polyclonal antibody responses. However, insect cell E2 induced stronger neutralizing antibody responses against the homologous isolate used in the vaccine, albeit the two proteins elicited comparable neutralization titers against heterologous isolates. A more productive approach for vaccine development may be complete deletion of specific glycans in the E2 protein.

## INTRODUCTION

Hepatitis C virus (HCV) is a major public health problem and infects over 71 million people worldwide (https://www.who.int/en/news-room/fact-sheets/detail/hepatitis-c). Infection often develops into chronic hepatitis, which is the most prevalent cause of liver failure and hepatocellular carcinoma. In recent years, new direct-acting antivirals (DAAs) have supplanted the use of ribavirin and pegylated interferon alpha as a treatment regimen, reaching cures rates of greater than 90% in HCV genotype 1-infected patients (1, 2). However, significant limitations still exist, including the high cost of DAAs that restricts access in developing countries, where the disease burden is greatest (2), and concerns about the development of drug resistance (1, 2). Moreover, therapy-induced HCV clearance does not provide immunity to new infections or eliminate the risk of hepatocellular carcinoma in patients with established cirrhosis. Therefore, an effective preventative vaccine is an important medical and public health need (3).

The genetic diversity of HCV of at least seven genotypes that differ up to 30% in nucleotide sequence poses a major challenge to developing a pangenotypic vaccine. HCV is composed of a nucleocapsid core enveloped by a lipid bilayer in which two surface glycoproteins, E1 and E2, are anchored. During acute infection, a robust neutralizing antibody response correlates with spontaneous resolution of infection (4, 5). Most broadly neutralizing antibodies (bNAbs) recognize conformational epitopes in E2 and some in E1E2 (6, 7). Passive immunization with anti-HCV antibodies before HCV challenge can prevent infection against homologous virus challenge in animal models (8, 9), although virus breakthrough can occur, and protection against heterologous virus strains remains a problem. Other complicating factors for vaccine development include low immunogenicity of viral envelope proteins and antibody responses directed to regions that display a high mutational rate of change (10). In addition, a number of studies have highlighted the role of *N*-linked glycosylation in masking or shielding E2 epitopes from recognition by bNAbs (11–15), as also observed for HIV and influenza virus (16). Such glycan shielding may hinder bNAb induction by reducing the exposure of neutralizing epitopes to the humoral immune system, further complicating the development of a B cell-based HCV vaccine.

There is evidence from a number of studies, mainly involving HIV and influenza virus, that alteration of glycans on viral envelope glycoproteins can impact immune recognition and immunogenicity. Some studies have used hyperglycosylation, the addition of *N*-glycans through mutagenesis to create glycan sequons, to mask certain sites associated with nonneutralizing antibodies and antibody escape (17–21). Conversely, targeted removal of glycans through mutagenesis of glycan sequons has been used to improve or alter neutralizing antibody engagement of key epitopes for HIV-1 (22–25), influenza A virus (26), and HCV (12–14). For example, in one study, the elimination of glycosylation sites in the vicinity of the CD4 binding site of the HIV-1 envelope glycoprotein (Env) generated an immunogen that more efficiently activated B cells expressing germ line precursors of bNAbs (24). However, in another study, targeted deglycosylation of HIV-1 Env failed to generate an antigen capable of eliciting neutralizing antibodies against wild-type viruses in immunized guinea pigs or rhesus macaques (27). In the case of influenza A virus, the removal of glycans in the hemagglutinin (HA) protein that shield key epitopes of HA from antibody recognition produced immunogens that elicited bNAbs effective against antigenically distant virus strains (26). In the case of HCV, the deletion of specific glycans in HCV E1E2 was found to modulate binding of certain bNAbs (13, 14), although the immunogenicity of these modified E1E2 proteins was not studied.

An alternate strategy to modulate the presentation of key antibody epitopes is modification of the extent of glycosylation through the use of various expression cell types (28–31), or enzymatic truncation of glycoforms (32). In one study, HIV-1 gp120 was produced in Spodoptera insect (Sf9) cells, which impart mainly paucimannose-type glycans, and in mammalian (HEK293) cells treated with the α-mannosidase inhibitor kifunensine, which confers only high-mannose-type glycans (29). These global alterations in glycosylation compared to gp120 produced in untreated HEK293 cells led to increased exposure of the CD4 binding site, which encouragingly translated to increased binding of bNAbs specific for this site. However, the effect on HIV-1 neutralization titers in animals immunized with these modified gp120 glycoproteins was not reported. More recently, a secreted soluble form of HCV E2 (sE2) produced in Drosophila insect (S2) cells was found to be more immunogenic than the corresponding protein produced in HEK293 cells (30). Moreover, S2-derived sE2 elicited higher titers of antibodies capable of neutralizing a diverse panel of HCV genotypes, suggesting that distinct glycosylation patterns should be taken into consideration in the development of a recombinant HCV vaccine.

To test further the hypothesis that differential glycosylation may influence the antigenicity and immunogenicity of E2, we performed head-to-head molecular, antigenic, and immunogenic comparisons of sE2 produced in (i) mammalian (HEK293) cells, which impart high-mannose, hybrid, and complex glycans; and (ii) insect (Sf9) cells, which confer mainly paucimannosidic glycans. In contrast to Li et al. (30), we found that immunization of mice with mammalian and insect sE2 glycoproteins elicited comparable antibody neutralization titers against heterologous HCV isolates, although Sf9-derived sE2 was a more potent immunogen against the homologous H77c isolate. Here, we discuss possible reasons for the apparent discrepancy between our results and theirs and conclude that targeted deletion of specific E2 glycans, rather than expression system-dependent modification of all glycans, may be a better strategy for increasing the exposure of virus-neutralizing epitopes to the humoral immune system.

## RESULTS

### Expression of soluble HCV E2 glycoprotein in HEK293 and Sf9 cells.

**FIG 1:**
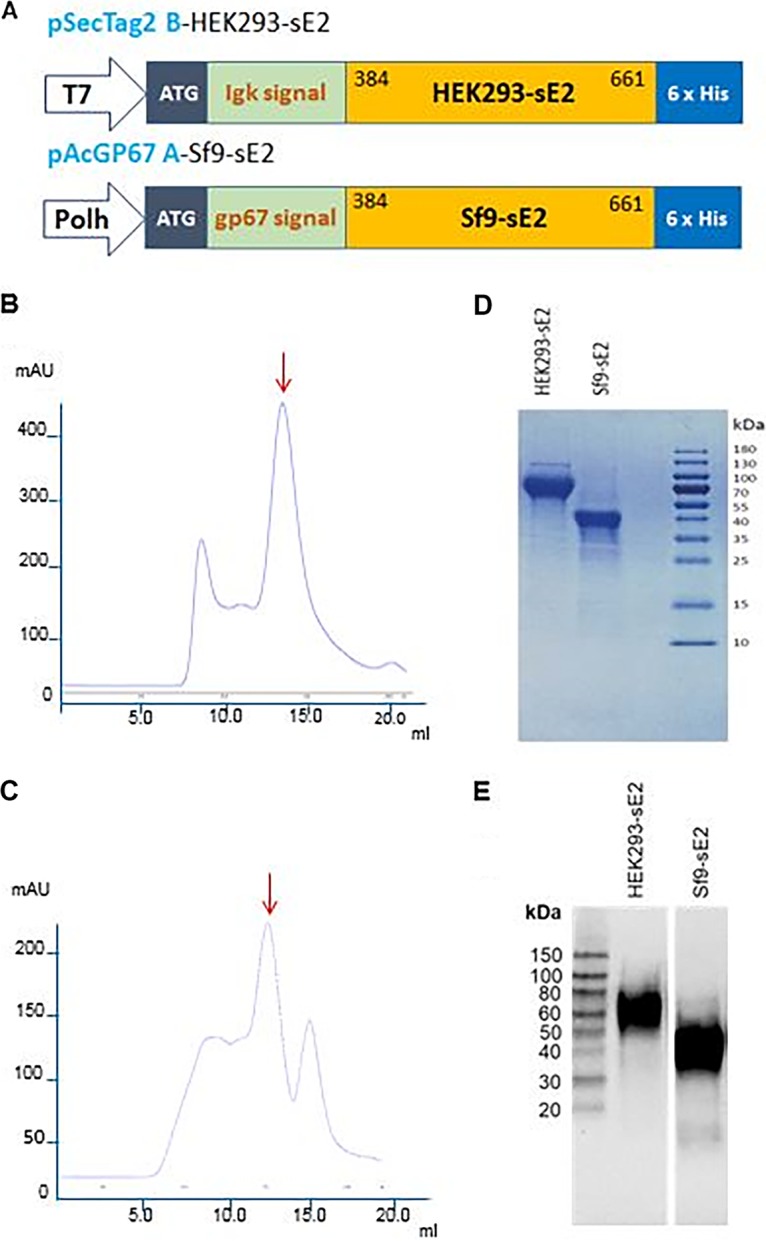
Expression of soluble HCV E2 (sE2) derived from mammalian (HEK293) and insect (Sf9) cells. (A) Schematic representation of sE2 expression cassettes used in mammalian (HEK293-sE2) and insect cell (Sf9-sE2) vectors. A sequence encoding sE2 (amino acids 384 to 661) from genotype 1a (H77) was cloned into the vector pSecTag2 in-frame with an immunoglobulin light-chain κ signal peptide sequence and fused with a C-terminal His_6_ tag. Recombinant plasmid was used to transfect HEK293-F cells. The same sequence was cloned into the baculovirus expression vector pAcGP67-B with a polyhedrin (Polh) promoter. The construct was transfected into Sf9 cells together with BaculoGold linearized baculovirus DNA to produce sE2. (B) Superdex 200 size-exclusion chromatography profile of HEK293-derived sE2 following HisTrap Ni^2+^-NTA affinity chromatography. Peak (red arrow) corresponding to monomeric sE2 was isolated for analytical and immunization studies. mAU, milliabsorbance units. (C) Superdex 200 chromatography profile of Sf9-derived sE2 following HisTrap Ni^2+^-NTA chromatography. Peak (red arrow) corresponding to monomeric sE2 was isolated for all further experiments. Peak marked by red arrow corresponds to monomeric sE2. (D) Nonreducing SDS-PAGE (10% gels) of purified HEK293- and Sf9-derived sE2 proteins used for analytical characterization and immunization studies (Coomassie blue G-250 staining). (E) Western blot analysis of purified HEK293- and Sf9-derived sE2 proteins. Proteins were separated on SDS-PAGE (4 to 15%) gels after reduction by β-mercaptoethanol and boiling for 10 min. Proteins were then transferred onto a nitrocellulose membrane (Bio-Rad Laboratories). The membrane was probed with anti-HCV E2 HMAb HC33.1 (6) at 4.5 μg/ml, followed by secondary goat anti-human IgG-horseradish peroxidase (IgG-HRP) conjugate at a 1:5,000 dilution.

In order to investigate the effect of different glycosylation patterns on the antigenicity and immunogenicity of HCV E2, we produced a soluble form of E2 (sE2) lacking the hydrophobic C-terminal transmembrane anchor in mammalian (HEK293) and insect (Sf9) cells, which are known to attach different *N*-glycan moieties to proteins (33, 34). For expression of sE2 in HEK293 cells, a DNA fragment encoding amino acids 384 to 661 of strain 1a HCV H77c polyprotein was cloned into the pSecTag2 vector under the control of the cytomegalovirus (CMV) promoter. The same DNA fragment was used for expression of sE2 in Sf9 insect cells using the pAcGP67-B vector, in which expression is controlled by the polyhedrin promoter (Fig. 1A). Secreted monomeric sE2 proteins bearing C-terminal His_6_ tags were purified from culture supernatants by immobilized Ni^2+^ affinity chromatography, followed by size-exclusion chromatography. Superdex 200 elution profiles for HEK293- and Sf9-derived sE2 showed that both proteins formed oligomers and high-molecular-weight aggregates, in addition to monomers (Fig. 1B and C). In both cases, we isolated the peak corresponding to monomeric sE2. Only monomeric HEK293- and Sf9-derived sE2 proteins were used for all subsequent analytical and immunological experiments. As shown in Fig. 1D, the HEK293- and Sf9-derived sE2 proteins migrated in nonreducing SDS-PAGE at apparent molecular weights of ∼75 kDa and ∼40 kDa, respectively, compared to a calculated molecular weight of ∼34 kDa based on primary amino acid sequence. Thus, sE2 is glycosylated differently by mammalian and insect cells, as expected (33, 34). In addition, we examined HEK293- and Sf9-derived sE2 in SDS-PAGE under reducing conditions and carried out Western blot analysis using human monoclonal antibody (HMAb) HC33.1 (Fig. 1E). HC33.1 recognizes a linear epitope of HCV E2 (domain E) comprising residues 412 to 423 (6). The Western blot analysis revealed major bands at ∼60 kDa and ∼40 kDa for reduced HEK293- and Sf9-derived sE2, respectively, which is similar to what was observed under nonreducing conditions (Fig. 1D). There was no indication of significant proteolysis of either HEK293- or Sf9-derived sE2 (Fig. 1E). Moreover, reactivity with HC33.1 confirmed the identity of both purified proteins as HCV E2. We therefore conclude that Sf9-derived sE2 is intact and not more susceptible to proteolysis than HEK293-derived sE2, despite differences in glycosylation. To pinpoint these differences, we used mass spectrometry (MS) to identify the nature of sugars attached to HEK293- and Sf9-derived sE2.

### *N*-Glycosylation analysis of HCV E2 expressed in HEK293 and Sf9 cells.

Three major types of mature *N*-glycans may be attached to glycoproteins such as HCV E2 produced in mammalian cells: (i) high-mannose glycans, consisting of up to six mannose (Man) residues attached to a trimannosyl chitobiose core (Man_3_GlcNAc_2_); (ii) complex-type glycans, composed primarily of *N*-acetylglucosamine (GlcNAc) and galactose (Gal) residues attached to the Man_3_GlcNAc_2_ core, with or without sialic acid (NeuAc) residues, where a fucose (Fuc) residue may be added to the first GlcNAc of the core; and (iii) hybrid-type glycans, which consist of Man and lactosamine (GlcNAcGal) residues, with or without NeuAc, attached to the Man_3_GlcNAc_2_ core (33, 34). Insect expression systems, on the other hand, commonly express paucimannose structures as well as core α1,3 fucosylation absent in mammalian expression systems.

**FIG 2:**
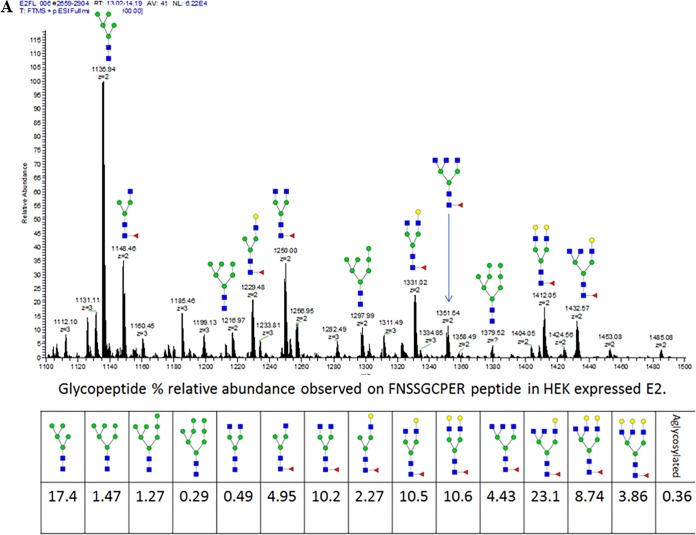

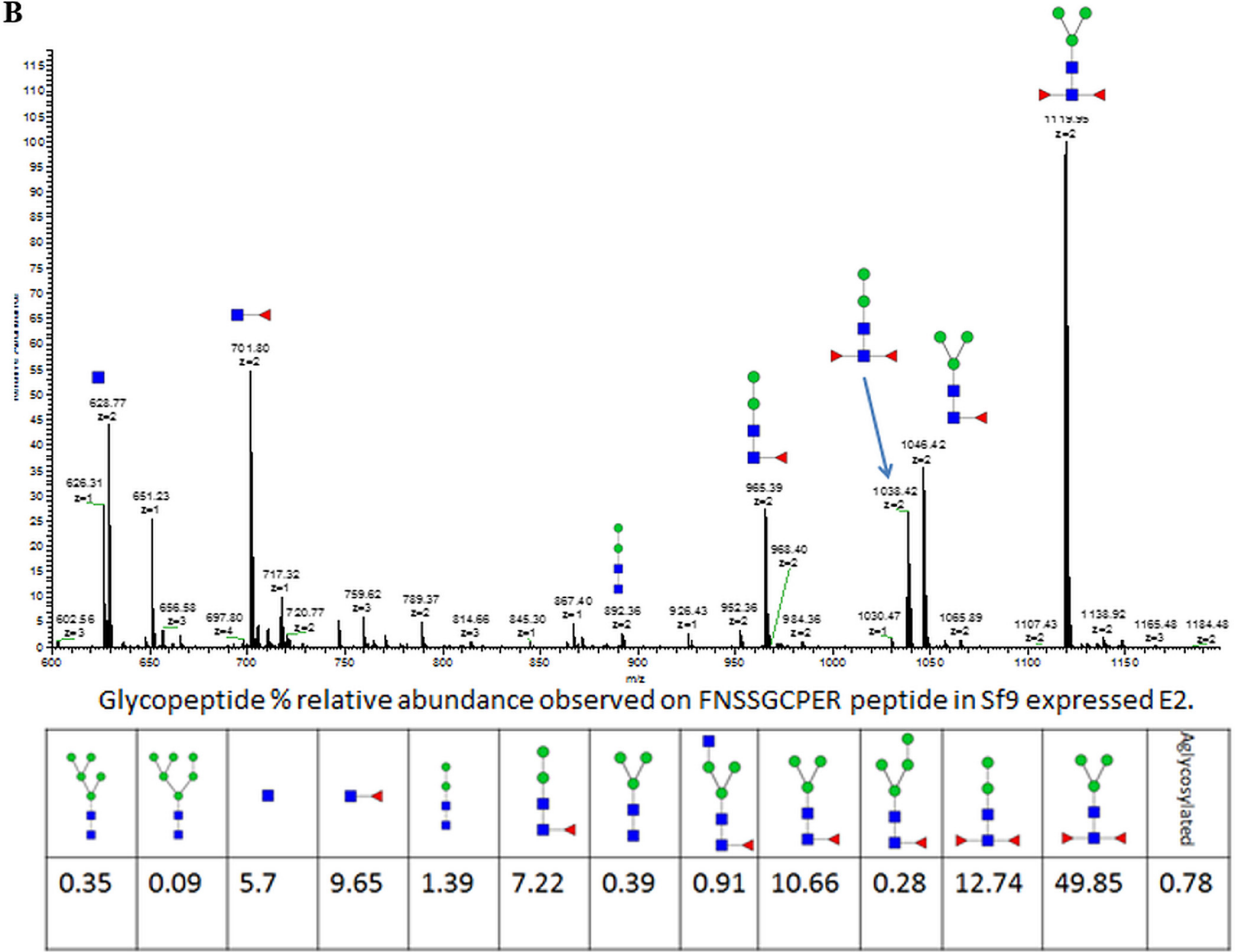
Representative electrospray ionization MS (ESI-MS) of chymotryptic peptide FNSSGCPER (amino acids 447 to 455) from HCV sE2. (A) Glycopeptide heterogeneity observed on FNSSGCPER peptide from HEK293-expressed sE2 (relative abundance [RA], ≥1% listed in spectrum and RA, ≥0.1% listed in table). (B) Glycopeptide heterogeneity observed on FNSSGCPER peptide in Sf9-expressed E2 (RA, ≥1% listed in spectrum and RA, ≥0.1% listed in table). Note that only doubly charged glycopeptides are labeled in the spectrum for clarity for both panels A and B.

We used MS to characterize the glycan moieties attached to the 11 potential *N*-glycosylation sites of HCV E2 derived from HEK293 and Sf9 cells. The composition and microheterogeneity of the glycans attached to each *N*-linked site were determined by liquid chromatography-UV-tandem MS (LC-UV-MS/MS) analyses of enzymatic digests (trypsin and chymotrypsin sequential digestion) of reduced and alkylated HEK293- and Sf9-derived sE2. Glycopeptide identifications were made based on MS/MS fragmentation, followed by relative abundance calculation using extracted ion chromatograms of the parent ion. Representative MS spectra of the glycopeptide at positions 447 to 455 of HEK293- and Sf9-derived sE2 are shown in Fig. 2. Doubly charged glycopeptides observed above 1% relative abundance are depicted to demonstrate the general trend of glycans of larger composition observed in HEK293-derived sE2. A complete list of the glycan compositions identified at each glycosylation site of HEK293-derived versus Sf9-derived sE2, as well as the corresponding glycan populations down to 0.1% relative abundance based on extracted ion chromatograms of the parent ions, is presented in Table S1 in the supplemental material. These 11 glycosylation sites are located at E2 positions 417 (designated N1), 423 (N2), 430 (N3), 448 (N4), 476 (N5), 532 (N6), 540 (N7), 556 (N8), 576 (N9), 623 (N10), and 645 (N11).

**FIG 3:**
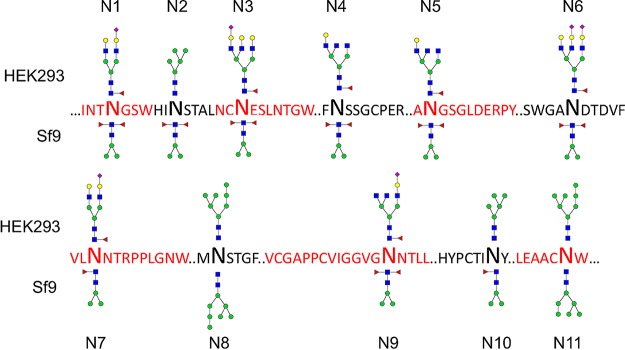
Schematic representation of HCV sE2 chymotryptic glycopeptides depicted with the most abundant glycan observed during LC-MS analysis of HEK293 (top glycoform) and Sf9 (bottom glycoform) sE2 proteins. In cases where the most abundant proteoform was aglycosylated, the most abundant glycosylated form was used (Table S1). “…” represents flanking amino acid sequence not shown for brevity. *N*-acetylglucosamine, blue squares; fucose, red triangles; mannose, green circles; galactose, yellow circles; sialic acid, purple diamonds.

Multiple complex- and high-mannose-type glycans were identified at 11 of 11 *N*-linked sites of HEK293-derived sE2 (Table S1). As expected for a glycoprotein expressed in insect cells (33, 34), the major glycoforms present on Sf9-derived sE2 were paucimannose *N*-glycans, with or without one or two core Fuc residues. Complex *N*-glycans were detected at all sites except N7 in Sf9-derived sE2, albeit with a lower degree of extension and branching on average than in HEK293-derived sE2. Complex-type glycans accounted for only 1% to 10% of the total identified *N*-glycans in Sf9-derived sE2 (Table S1). The most abundant glycoform at each site observed in LC-MS analysis of HEK293- and Sf9-derived sE2 is depicted in Fig. 3.

**FIG 4:**
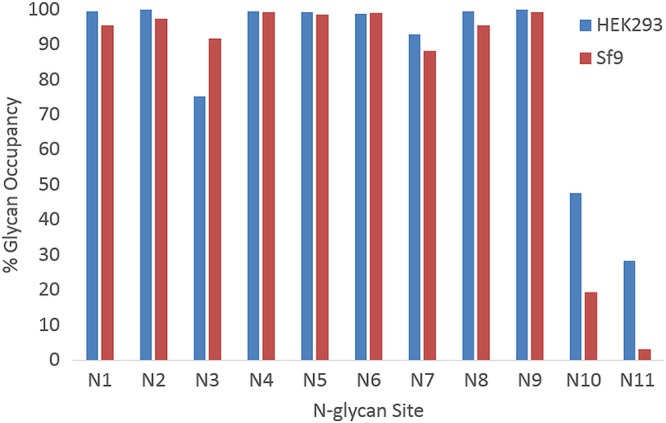
Comparison of percent glycosylation of *N*-glycan sites for mammalian cell-expressed (HEK293) and insect cell-expressed (Sf9) sE2 glycoproteins. Most *N*-glycan sites are fully or nearly fully glycosylated, with the exception of N10 (N623) and N11 (N645), where *N*-glycan sites have relatively lower glycan occupancy for Sf9-derived eE2, whereas glycan N3 (N430) has lower glycan occupancy in HEK293 cells.

For both HEK293- and Sf9-derived sE2, the same nine *N*-glycan sites are near fully (>95% for N1, N2, N4, N5, N6, N8 and N9) or highly (>75% for N3 and N7) glycosylated (Fig. 4). Of note, the two exceptions (N10 and N11) exhibit relatively low levels of glycan occupancy in both mammalian and insect cell-derived sE2. This concordance in the extent of glycosylation for sE2 from two hosts with significantly different protein *N*-glycosylation pathways suggests that factors intrinsic to E2 itself, such as differential accessibility of individual glycosylation sites to the cellular *N*-glycosylation biosynthetic machinery, may explain the observed site-specific differences in percent glycosylation (Fig. 4).

### Molecular modeling of *N*-glycosylation sites.

**FIG 5:**
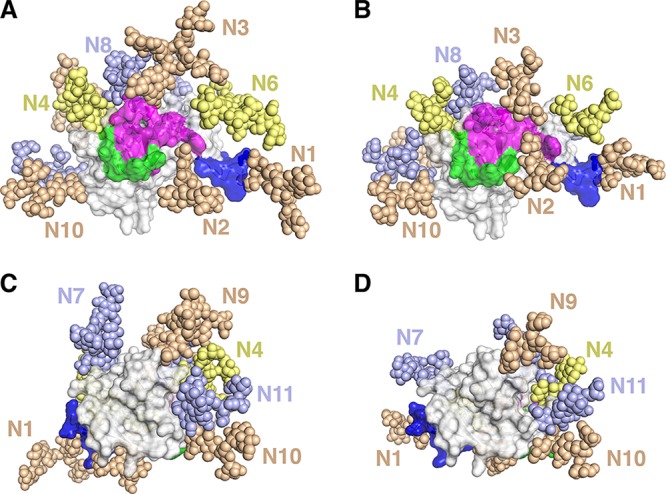
Modeled structural impact of glycosylation for HEK293- versus Sf9-expressed sE2. Structures of the most abundant glycoforms for HEK293 (A and C) and Sf9 (B and D) sE2 were modeled using Rosetta (52) onto the E2 core crystal structure (PDB ID 4MWF) (41). Glycans are shown as tan, slate, and yellow sticks and labeled, and E2 is shown as surface and cartoon, with views of E2 front layer (A and B) and back layer (C and D). For reference, neutralizing antibody footprints on E2 are colored blue, magenta, and green, based on the epitope-bound crystallographic structures of HC33.1 (PDB code 4XVJ), AR3C (PDB code 4MWF), and HC84.26.5D (PDB code 4Z0X) antibodies, respectively. Colored E2 residues indicate those within 5.0 Å of bound antibody, and shared E2 contact residues between antibodies are colored according to antibody with highest E2 residue burial. Residues from antigenic domain E were modeled at the N terminus of the E2 core structure using Rosetta (52). Glycan N5, which is within a region that is missing from the E2 core crystal structure, is not shown.

To compare the structural impact of *N*-glycosylation in sE2 derived from HEK293 versus Sf9 cells, we modeled the most abundant glycoform at each site on the E2 core structure (Fig. 5). This analysis highlighted the differential glycan sizes observed in the two expression systems, particularly those proximal to key epitopes such as N6 (Fig. 5A and B), which is adjacent to the AR3C binding site in antigenic domain B (6) and is markedly larger for HEK293- versus Sf9-expressed sE2. These models were used to calculate protein surface occlusion from glycosylation (Table 1). Though a solvent-sized probe (1.4-Å radius) detected little difference between E2 surface occlusion in the models, larger probe radii (5 and 10 Å), which are more representative of antibody complementarity-determining region (CDR) loops, revealed relatively high E2 occlusion (40% to 65% of the protein surface), with HEK293-derived sE2 having somewhat higher levels of masked surface than Sf9-derived sE2 (9% higher for 10-Å probe). This is similar to previous observations comparing glycosylation of HEK293- versus Sf9-expressed HIV gp120, where approximately 10% greater surface occlusion for HEK293-expressed gp120 was calculated using a 10-Å probe radius (29). However, it should be noted that this analysis does not include E2 regions missing from the crystal structure (e.g., hypervariable region 1 [HVR1]), E2 dimerization with E1, and conformational mobility of glycans and E2 residues. Nevertheless, the calculated 9% reduction in protein surface coverage imparted by the Sf9 cell production system could potentially affect the exposure of antibody binding sites proximal to glycans.

**TABLE 1:** Calculated E2 percent surface occlusion by most abundant *N*-glycans from HEK293 and Sf9 expression systems modeled onto the E2 core structure

Probe radius (Å)	% surface occlusion in:	
	HEK293	Sf9
1.4	20	21
5	49	43
10	64	55

### CD81 binding and antigenicity of HCV E2 expressed in mammalian versus insect cells.

**FIG 6:**
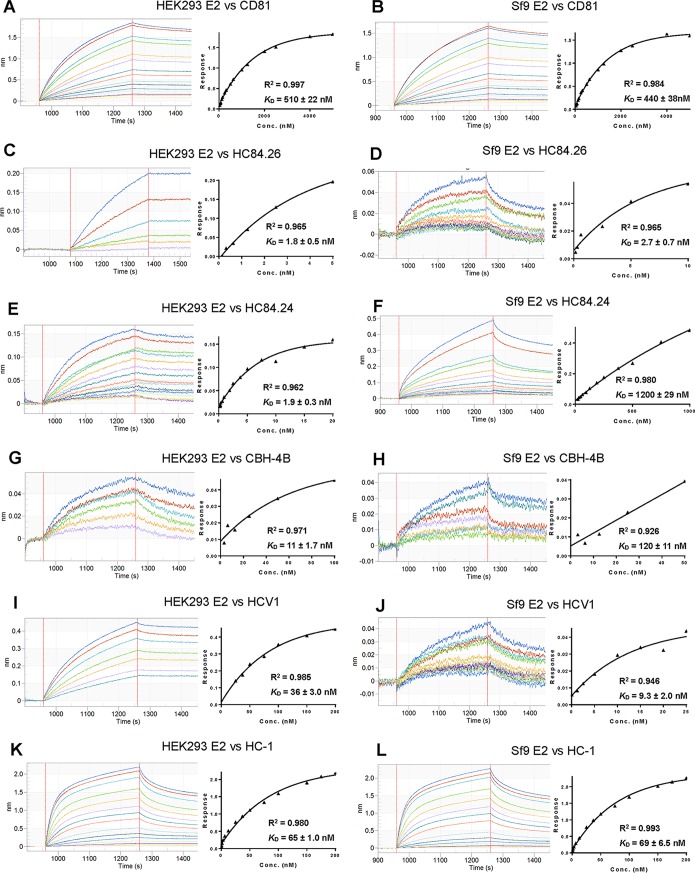
BLI analysis of CD81 and antibody binding to HCV sE2 from HEK293 and Sf9 expression systems. (A) Sensograms (left) for CD81 binding to immobilized HEK293-derived sE2. CD81 concentrations were 5,000, 4,000, 2,500, 2,000, 1,250, 1,000, 625, 500, 312.5, 250, 156.25, 125, 78.125, 62.5, and 39.06 nM. Steady-state analysis graph (right) gave a *K_D_* of 510 ± 22 nM. (B) Sensograms (right) for CD81 binding to immobilized Sf9-derived sE2. CD81 concentrations were 5,000, 4,000, 2,500, 2,000, 1,250, 1,000, 625, 500, 312.5, 250, 156.25, 125, 78.125, 62.5, and 39.06 nM. Steady-state analysis graph (right) gave a *K_D_* of 440 ± 38 nM. (C) Sensograms (left) for HC84.26 (domain D-specific HMAb) binding to immobilized HEK293-derived sE2. HC84.26 concentrations were 5, 2.5, 1.25, 0.625, 0.3125, and 0.156 nM. Steady-state analysis graph (right) gave a *K_D_* of 1.8 ± 0.5 nM. (D) Sensograms (left) for HC84.26 binding to immobilized Sf9-derived sE2. HC84.26 concentrations were 10, 5, 2.5, 0.625, 0.3125, and 0.156 nM. Steady-state analysis graph (right) gave a *K_D_* of 2.7 ± 0.8 nM. (E) Sensograms (left) for HC84.24 (domain D-specific HMAb) binding to immobilized HEK293-derived sE2. HC84.24 concentrations were 20, 15, 10, 7.5, 5, 3.75, 2.5, 1.875, 0.9375, 0.625, 0.469, 0.3125, 0.234, and 0.156 nM. Steady-state analysis graph (right) gave a *K_D_* of 1.9 ± 0.3 nM. (F) Sensograms (left) for HC84.24 binding to immobilized Sf9-derived sE2. HC84.24 concentrations were 1,000, 750, 500, 375, 250, 187.5, 125, 93.8, 62.5, 46.9, 31.6, and 23.4 nM. Steady-state analysis graph (right) gave a *K_D_* of 1,200 ± 290 nM. (G) Sensograms (left) for CBH-4B (domain A-specific HMAb) binding to immobilized HEK293-derived sE2. CBH-4B concentrations were 100, 50, 25, 12.5, 6.25, and 3.125 nM. Steady-state analysis graph (right) gave a *K_D_* of 11 ± 1.7 nM. (H) Sensograms (left) for CBH-4B binding to immobilized Sf9-derived sE2. CBH-4B concentrations were 50, 25, 12.5, 6.25, and 3.13 nM. Steady-state analysis graph (right) gave a *K_D_* of 120 ± 11 nM. (I) Sensograms (left) for HCV1 (domain E-specific HMAb) binding to immobilized HEK293-derived sE2. HCV1 concentrations were 200, 150, 100, 75, 50, 37.5, and 25 nM. Steady-state analysis graph (right) gave a *K_D_* of 36 ± 3.0 nM. (J) Sensograms (left) for HCV1 binding to immobilized Sf9-derived sE2. HCV1 concentrations were 25, 20, 15, 10, 5, 2.5, 1.25, and 0.078 nM. Steady-state analysis graph (right) gave a *K_D_* of 9.3 ± 2.0 nM. (K) Sensograms (left) for HC-1 (domain B-specific HMAb) binding to immobilized HEK293-derived sE2. HC-1 concentrations were 200, 175, 150, 75, 50, 37.5, 25, 12.5, 6.25, 3.125, 1.56, 0.78, 0.39, and 0.195 nM. Steady-state analysis graph (right) gave a *K_D_* of 65 ± 10 nM. (L) Sensograms (left) for HC-1 binding to immobilized Sf9-derived sE2. HC-1 concentrations were 200, 175, 150, 75, 50, 37.5, 25, 12.5, 6.25, 3.125, 1.56, 0.78, 0.39, and 0.195 nM. Steady-state analysis graph (right) gave a *K_D_* of 69 ± 6.5 nM.

To determine whether differential glycosylation affected binding to the CD81 entry receptor, we used biolayer interferometry (BLI) to measure the affinity of HEK293- and Sf9-derived sE2 for recombinant CD81 (Fig. 6A and B). Purified sE2 proteins were directionally coupled to a Ni^2+^-nitrilotriacetic acid (Ni^2+^-NTA) biosensor surface through their C-terminal His_6_ tags. HEK293-derived sE2 bound CD81 with an absolute measured affinity (*K_D_*) of 510 nM compared to 440 nM for Sf9-derived sE2 (Table 2). These very similar *K_D_* values demonstrate that both glycoproteins are functional with respect to entry receptor binding. Moreover, this result indicates that the proteins are properly folded since alanine-scanning mutagenesis has shown that the CD81 binding site comprises residues from several noncontiguous segments of the E2 polypeptide chain (i.e., it is conformational in nature) (6, 7).

**TABLE 2:** Affinities of HEK293 sE2 and Sf9 sE2 for CD81 and HMAbs

Ligand	HEK293 sE2		Sf9 sE2	
	*K_D_* (nM)	*R*^2^	*K_D_* (nM)	*R*^2^
CD81	510 ± 22	0.997	440 ± 38	0.984
HC84.26^*a*^	1.8 ± 0.5	0.965	2.7 ± 0.8	0.965
HC84.24^*a*^	1.9 ± 0.3	0.962	1,200 ± 29	0.980
CBH-4B^*b*^	11 ± 1.7	0.971	120 ± 11	0.926
CBH-4G^*b*^	120 ± 33	0.949	No binding	NA^*c*^
HCV1^*d*^	36 ± 3.0	0.985	9.3 ± 2.0	0.946
HC33.1^*d*^	50 ± 9.9	0.979	47 ± 9.9	0.927
HC-1^*e*^	65 ± 1.0	0.980	69 ± 6.5	0.993
HC-11^*e*^	7.0 ± 1.3	0.975	25 ± 4.4	0.974

a Specific for antigenic domain D.

b Specific for antigenic domain A.

c NA, not applicable.

d Specific for antigenic domain E.

e Specific for antigenic domain B.

To determine whether differential glycosylation affected epitope presentation, we used BLI to measure the binding of HEK293- and Sf9-expressed sE2 to a panel of eight human monoclonal antibodies (HMAbs) targeting the following four antigenic domains on E2: CBH-4B and CBH-4G (domain A), HC-1 and HC-11 (domain B), HC84.26 and HC84.24 (domain D), and HCV1 and HC33.1 (domain E) (6, 35) (Fig. 6C to L). Of note, this provides a direct comparative view of HEK293- versus Sf9-expressed sE2, while absolute measured affinity (*K_D_*) values may reflect effects of bivalent IgG binding. Five of these HMAbs bound both sE2 proteins with very similar *K_D_* values, all in the nanomolar range, as follows: HC84.26, 1.8 nM and 2.7 nM; HCV1, 36 and 9.3 nM; HC33.1, 50 and 47 nM; HC-1, 65 and 69 nM; and HC-11, 7.0 and 25 nM for HEK293-derived sE2 and Sf9-derived sE2, respectively (Table 2). Importantly, the ability of conformation-dependent HMAbs HC84.26, HC-1, and HC-11 (6) to recognize both versions of sE2 with high affinity indicates that both glycoproteins are correctly folded, in agreement with our CD81 binding results. The other three HMAbs bound HEK293-derived sE2 more tightly than Sf9-derived sE2, as follows: HC84.24, 1.9 nM and 1,200 nM; CBH-4B, 11 and 120 nM; and CBH-4G, 120 nM and no apparent binding for Sf9-derived sE2, respectively (Table 2). We attribute these affinity differences to differential glycosylation, given that glycans cover ∼50% of the surface of sE2 (Fig. 5) and so are likely to influence HMAb binding to at least some epitopes, either directly or indirectly (see Discussion). These results demonstrate that increasing protein surface exposure by reducing glycan size did not translate into enhanced antigenicity, at least for the eight HMAbs tested here.

### Immunization of mice with sE2 induces serum antibodies that recognize broadly neutralizing epitopes.

**FIG 7:**
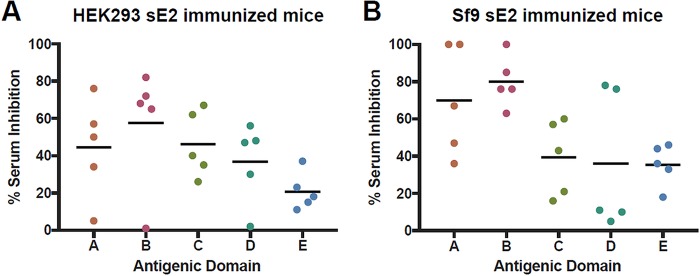
Competition binding analysis of immune sera corresponding to domains A to E. (A) HEK293-derived sE2 immunized CD-1 mice. (B) Sf9-derived sE2 immunized CD-1 mice. Sera from day 42 were tested for binding competition using antibodies representing antigenic domains A to E (6), with mean percent competition shown with black bars. All values were normalized by subtracting percent competition values from control (preimmune) sera, which were measured individually for each mouse. Antibodies tested were CBH-4B (domain A), HC-1 (domain B), CBH-7 (domain C), HC84.26 (domain D), and HC33.4 (domain E).

To compare the antibody responses in mice immunized with the HEK293- and Sf9-derived sE2 proteins, groups of CD-1 mice (*n* = 5) were immunized with 50 μg of purified protein formulated in SAS adjuvant (oil-in-water emulsion), followed by four 10-μg boosts over a course of 5 weeks. Serum samples were collected 1 week after the fifth immunization (day [D] = 42), and sera were tested for epitope-specific responses by competitive binding enzyme-linked immunosorbent assay (ELISA) using HMAbs derived from HCV-infected individuals that represent the following five antigenic domains of E2 (6–9): CBH-4B (domain A), HC-1 (domain B), CBH-7 (domain C), HC84.26 (domain D), and HC33.3 (domain E) (Fig. 7). Based on binding competition, antibodies were elicited to all five antigenic domains in both groups of mice. Both groups had relatively high levels of serum competition with the antigenic domain B HMAb HC-1; this domain is associated with broadly neutralizing antibodies and overlaps the CD81 binding site (6). Comparing the two groups of immunized mice, no significant differences were observed for domain-specific serum antibody competition (*t* test, GraphPad Prism 7).

### Viral neutralization against homologous and heterologous isolates of HCV.

**FIG 8:**
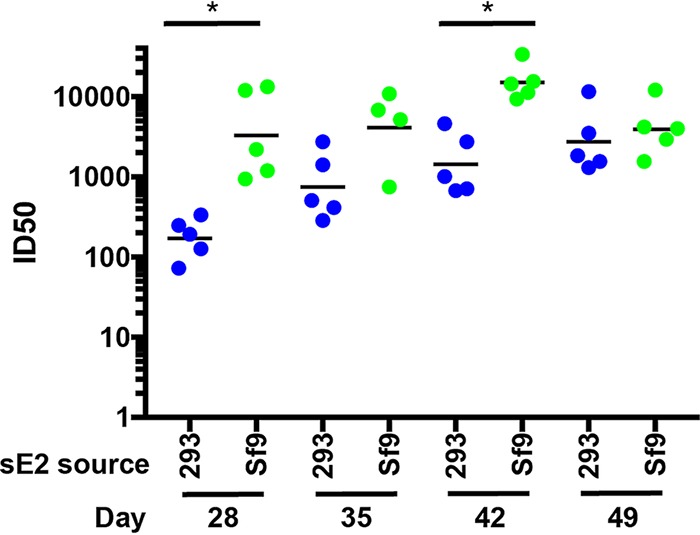
Kinetics and magnitude of nAb titers against homologous H77 strain. HCV pseudoparticles (HCVpps) were generated by cotransfection of HEK293T cells with HCV E1E2, MLV Gag-Pol packaging vector, and luciferase reporter plasmid, as previously described (36). Titrations of HCVpp were performed on Hep3B cells, and luciferase activity measured in relative light units (RLUs) using SpectraMax M3. Percent neutralization was calculated as 100 × [1 − *y*RLUtest/RLUcontrol]. nAb titers in animal sera are reported as 50% inhibitory dilution (ID_50_) values, calculated using nonlinear curve fitting in GraphPad Prism. Neutralization kinetics were determined by inhibition of homologous HCVpp (H77) using serial dilutions of serum collected on days 28, 35, 42, and 49. Blue dots represent serum samples from HEK293 sE2-immunized mice, green dots represent samples from Sf9 sE2-immunized mice, and black bars indicate geometric means. One Sf9 E2-immunized mouse had insufficient day 35 serum available for testing; thus, four rather than five points are shown for that group and day. *P* values were determined using GraphPad Prism 7 with Kruskal-Wallis ANOVA, and significant *P* values between immunized groups are indicated (*, *P* < 0.05).

In order to assess the relative potencies of the HEK293- and Sf9-derived sE2 immunogens, mouse sera were tested for neutralizing antibody (nAb) titers against homologous and heterologous isolates of HCV E1E2 using the HCV pseudoparticle (HCVpp) neutralization assay. The values are expressed as serum dilution levels corresponding to 50% neutralization (ID_50_). As shown in Fig. 8, the mean ID_50_ values of nAb against the homologous isolate (H77c) (i.e., same E1E2 sequences as used in the vaccine) were 5- to 10-fold higher for Sf9-derived versus HEK293-derived sE2 after the third (D = 28), fourth (D = 35), and fifth (D = 42) immunizations, with significant differences between groups at days 28 and 42. However, the difference diminished 2 weeks after the last immunization (D = 49) to give approximately the same values.

**FIG 9:**
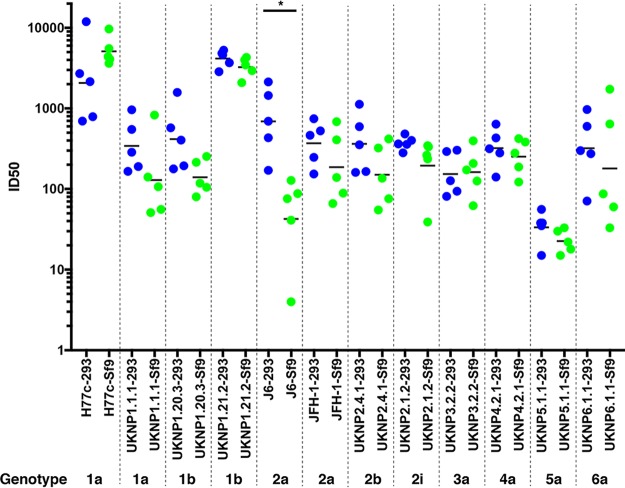
Breadth of neutralization against HCV genotypes 1 to 6. HCVpps generated with functional E1E2 clones derived from six diverse HCV genotypes, gt1a (H77c, UNKP1.1.1), gt1b (UNKP1.20.3, UNKP1.21.2), gt2a (J6, JFH), gt2b (UNKP2.4.1), gt2i (UNKP2.1.2), gt3 (UNKP3.2.2), gt4 (UNKP4.2.1), gt5 (UNKP5.1.1), and gt6 (UNKP6.1.1), were assessed for their neutralization sensitivity (50% neutralization titer [ID_50_]) to CD-1 mouse serum samples immunized with HEK293-derived sE2 (blue dots) and Sf9-derived sE2 (green dots), using HuH7 target cells. Geometric mean ID_50_ values are shown as black bars. *P* values were determined using GraphPad Prism 7 with Kruskal-Wallis ANOVA, and significant *P* values between immunized groups are indicated (*, *P* < 0.05).

To assess the breadth of neutralization, mouse sera (day 49) were subsequently tested against a diverse panel of six HCV genotypes (gt), gt1a, gt1b, gt2a, gt2b, gt2i, gt3a, gt4a, gt5a, and gt6a, as previously described (36). As shown in Fig. 9, animals immunized with both HEK293- and Sf9-derived sE2 were able to elicit bNAbs against all of the HCVpps tested in the panel, although genotypes 1a and 1b had the highest ID_50_ titers overall. The ID_50_ values against the homologous H77c isolate were moderately, though not significantly, higher for Sf9-derived sE2. This confirms the relative H77c neutralization titers observed at this time point shown in Fig. 8, which was performed separately using different target cells (Hep3B cells, versus HuH7 cells in Fig. 9). For most of the other isolates, there were modest, though insignificant, increases in ID_50_s for the HEK293 sE2-immunized group, with the exception of the J6 isolate, for which a significant increase in the HEK293 sE2 titers was observed. It is possible that this modest decrease in breadth could be due to a more pronounced nAb response to HVR1 or other variable regions induced by Sf9-expressed sE2 versus HEK293-expressed sE2. HVR1 has been reported as an immunodominant site for nAbs, although the nAb response is largely strain specific (37–40). Therefore, the lower level of ID_50_ values for the other heterologous HCVpp genotypes may reflect lack of nAb cross-recognition of the HVR1 sites due to sequence variation in this region.

## DISCUSSION

In this study, we asked whether reducing the overall glycosylation of HCV sE2 (gt1a, H77) by producing this protein in Spodoptera insect (Sf9) cells improved its antigenicity and/or immunogenicity compared to sE2 produced in mammalian (HEK293) cells. Mass spectrometry showed that all 11 predicted *N*-glycosylation sites were utilized in sE2 from both HEK293 and Sf9 cells. Although most sites were fully or nearly fully glycosylated, two sites (N10 and N11) showed low (<50%) glycan occupancy. As expected, *N*-glycans in insect sE2 were, on average, smaller and less complex than *N*-glycans in mammalian sE2.

After modeling the most abundant glycoform at each site on the E2 structure (41), we quantified glycan coverage by calculating the amount of protein surface exposed to a spherical probe the size of an antibody binding site. Our analysis revealed that 64% of the surface of HEK293-derived sE2 was occluded by *N*-glycans compared to 55% for Sf9-derived sE2, corresponding to a 9% reduction in glycan shielding in insect cell-expressed material. However, this reduction was not associated with improved HMAb binding (Table 2). The ability of CD81, HC84.26, HCV1, HC33.1, HC-1, and HC-11 to bind HEK293- and Sf9-derived sE2 equally well indicates that E2 recognition by these six ligands does not require complex-type glycans.

In contrast, HC84.24, CBH-4B, and CBH-4G bound Sf9-derived sE2 less well than HEK293-derived sE2. It is not particularly surprising that some HMAbs bind these two glycoproteins with different *K_D_* values given that (i) Sf9- and HEK293-derived sE2 are glycosylated quite differently; (ii) glycans cover ∼50% of the surface of E2 (Fig. 5) and so are likely to influence HMAb binding to at least some epitopes, either directly by forming part of such epitopes or indirectly by being proximal to them; (iii) the glycosylation profile of natural E2 (i.e., E2 produced in HCV-infected human liver cells) is probably more similar to that of HEK293-derived sE2 than of Sf9-derived sE2; and (iv) the HMAbs used in this study were derived from individuals naturally infected with HCV. A previous global epitope mapping study (6) found that mutation of certain E2 glycan sites, including glycan N10, which is located in the E2 back layer near the mapped CBH-4B and CBH-4G binding sites (Fig. 5) and shows reduced occupancy for Sf9-expressed sE2 (Fig. 4), affects E2 binding for these and other HMAbs. More revealing with respect to structural and functional integrity is the ability of Sf9- and HEK293-derived sE2 to bind the CD81 receptor with nearly identical *K_D_* values (Fig. 6A and B).

As observed for recombinant HIV-1 envelope trimer produced in HEK293 cells (22), our LC-UV-MS/MS analysis of HEK293-derived sE2 revealed the presence of underprocessed *N*-glycans that remain in oligomannose form (Man_5–9_GlcNAc_2_), possibly because the high density of glycans on the E2 surface imposes steric constraints that limit the actions glycan-processing enzymes in the Golgi compartment. We detected high-mannose *N*-glycans at all 11 *N*-linked sites of HEK293-derived sE2. However, except at sites N8 and N11, the large majority of total identified glycoforms at the other nine *N*-linked sites were complex-type glycans, which are a hallmark of passage through the Golgi apparatus. In this respect, the overall glycosylation profile of HEK293-expressed sE2 resembles that of E1E2 heterodimers associated with HCV pseudoparticles (HCVpps), which also display a majority of complex-type glycans (15, 42). In contrast, E1E2 heterodimers associated with cell culture-derived HCV (HCVcc) contain a more balanced mixture of high-mannose and complex-type glycans. This difference suggests that both HEK293-expressed sE2 and HCVpp-associated E1E2 are more accessible to Golgi glycan-processing enzymes than are HCVcc-associated E1E2, possibly because, in the HCVcc system, the E1E2 glycoprotein is associated with nascent viral particles as it travels through the secretory pathway, thereby restricting its exposure to Golgi enzymes.

In a previous study of HCV sE2 produced in mammalian (CHO) cells (43), complex glycans were detected at only 2 of 11 *N*-linked sites (N2 and N3); all 9 other sites were occupied by high or paucimannose *N*-glycans. In contrast, we identified complex glycans at all 11 *N*-linked sites. Moreover, a complex glycan was the most abundant glycoform at 6 of these 10 sites (N1, N3, N4, N5, N6, and N9) (Fig. 3). These surprisingly large differences in glycosylation profiles may result from the use of different mammalian cell lines for sE2 expression, with HEK293 used here versus CHO in the earlier study (43). The substantially higher content of complex *N*-glycans in HEK293-derived sE2 indicates more extensive processing of oligomannoses during passage of this particular glycoprotein through the Golgi compartment of HEK293 than CHO cells.

We found that differences in *N*-glycosylation of sE2 produced in HEK293 versus Sf9 cells did not appreciably alter recognition by most HMAbs. Immunogenicity studies in mice showed that similar polyclonal antibody responses were elicited against domains A to E by competition binding analysis. Neutralizing antibody titers showed that Sf9-derived sE2 was a more potent immunogen than HEK293-derived sE2 when tested against the homologous H77c isolate. However, both HEK293- and Sf9-derived sE2 elicited robust neutralizing antibody titers when tested against a diverse panel of HCVpp genotypes, although the overall titers trended higher for HEK293-derived sE2.

Our results differ markedly from those reported previously in a comparison of HEK293- and Drosophila S2-derived sE2 (30). In that study, the insect material was found to be a more potent immunogen against a diverse panel of heterologous isolates. Whether or not there is a difference in glycosylation between E2 proteins derived from Drosophila versus Spodoptera cells that could affect immunogenicity is an open question. At present, most knowledge of insect glycobiology comes from the Drosophila model (34), and it cannot be excluded that glycosylation in Spodoptera spp. may differ in certain aspects. However, our LC-UV-MS/MS analysis of Sf9-expressed sE2 revealed a glycosylation pattern dominated by paucimannose *N*-glycans, with or without one or two core fucose residues, which is entirely consistent with what would be expected for sE2 produced in S2 cells (33, 34). Therefore, it is unlikely that differences in *N*-glycosylation can explain the apparent ability of S2-derived sE2, but not Sf9-derived sE2, to elicit higher titers of bNAbs against diverse HCV genotypes than HEK293-derived sE2 (30).

Several other factors may be responsible for the discrepancy between our results and those of Li et al. (30). One is the different mouse strains used here (CD-1) and in the Li et al. study (BALB/c). Although CD-1 mice are outbred and have more genetic diversity than do inbred BALB/c mice, this likely did not contribute to the differences seen between the two studies because in this study, the B cell responses to sE2 are similar using CD-1 and BALB/c mice (data not shown). It is also possible that the different adjuvants used by us (SAS) and Li et al. (30) (alum/CpG) significantly affected the immune response to otherwise very similar insect cell-derived sE2 glycoproteins. For example, in a study comparing enzymatically demannosylated HIV-1 gp120 produced in CHO cells with its untreated counterpart, demannosylation was found to increase immunogenicity in an adjuvant-dependent manner. Thus, when administered in alum, demannosylated gp120 was more immunogenic than the untreated glycoprotein, but this difference disappeared when Quil-A, a saponin-based adjuvant, was used (44). In this study, we used SAS as the adjuvant, which is an oil-in-water emulsion, compared to alum/CpG used by Li et al. (30). It has been previously reported that innate and adaptive immune responses can differ between the two adjuvant systems depending on the antigen used, route of administration, and schedule (45). Nevertheless, both adjuvant systems are capable of eliciting robust B cell responses, so this also may not account for the breadth of neutralization differences seen between the two studies. Another difference with the Li et al. (30) study and ours is that our sE2 protein was derived from the gt1a isolate, H77, while their sE2 was from the gt1b isolate, Con1b. These have 22% sequence divergence at the amino acid level, which may impact the immunogenicity of specific epitopes or E2 overall; recent studies have demonstrated that HVR1 (amino acid divergence is 59% in that region between H77 and Con1b, with 16 out of 27 amino acids changed) and other E2 sites are responsible for differences in neutralization sensitivity for sets of E2-targeting bNAbs (46, 47). Finally, we tested neutralization using HCVpp, whereas Li et al. (30) used HCVcc. This difference is potentially significant because, as noted above, HCVpp and HCVcc have different glycosylation profiles (15, 42). Any one or any combination of the above-mentioned differences could account for the discrepancies between the two studies.

While conceptually very appealing, the idea that altering glycans on viral envelope glycoproteins can actually yield superior immunogens for vaccine development remains to be demonstrated, despite considerable effort by a number of laboratories and some promising results. For example, although HIV-1 gp120 produced in Sf9 cells displayed increased binding to antibodies targeting the CD4 binding site (29), the new generation of HIV-1 envelope trimers for vaccine use is expressed in mammalian, not insect, cells (48). Similarly, antigens based on HIV-1 envelope glycan deletion mutants have proven to be less effective than, or at best equivalent to, wild-type antigens at generating neutralizing antibody responses possessing both breadth and potency (16, 27, 49, 50). At present, selective deglycosylation of HIV-1 envelope trimers is under investigation as a strategy for activating naive B cells expressing germ line precursors of antibodies that target the CD4 binding site (24, 25). Given our findings that global alteration of HCV E2 glycosylation by expression in different cellular hosts did not appreciably affect antigenicity or overall immunogenicity, future efforts will focus on complete deletion, rather than only modification, of specific *N*-glycans in order to increase the exposure of virus-neutralizing epitopes on E2 to the humoral immune system. For example, the removal of *N*-glycans at E2 positions 417 (N1) and 532 (N6) has led to increased sensitivity of the mutant isolates to neutralization by MAbs to antigenic domains E and B, respectively (13, 14). A focused effort to eliminate these and other glycans that affect antibody binding to domains B, D, and E might lead to an improved immunogen to elicit their associated bNAbs.

## MATERIALS AND METHODS

### Protein expression, purification, and antibodies.

For mammalian cell expression, a gene encoding HCV E2 from strain 1a H77c (residues 384 to 661) was cloned into the vector pSecTag2 (Invitrogen) with an N-terminal immunoglobulin κ light-chain signal sequence (for secretion) and a C-terminal His_6_ tag (for purification). The construct was transfected with 293fectin into FreeStyle 293-F cells (Invitrogen). Recombinant monomeric E2 was purified from culture supernatants by sequential HisTrap Ni^2+^-NTA and Superdex 200 columns (GE Healthcare). For insect cell expression, the same E2 sequence was fused to the gp67 secretion signal sequence of baculovirus vector pAcGP67-B (BD Biosciences) with a C-terminal His_6_ tag. To generate recombinant baculovirus, this construct was transfected into Sf9 cells together with BaculoGold linearized DNA (BD Biosciences). Soluble monomeric E2 was purified from culture supernatants of Sf9 cells infected with recombinant baculovirus using sequential HisTrap Ni^2+^-NTA and Superdex 200 columns.

Human CD81 large extracellular loop (CD81-LEL; CD81 residues 113 to 201) coding sequence DNA was synthesized (GenScript) and cloned into the vector pHLsec (Addgene plasmid #99845; a gift from E. Yvonne Jones) which includes a C-terminal His_6_ tag. The construct was transfected with PEI MAX 40K (Polysciences) into FreeStyle HEK293-F cells. CD81-LEL was purified from culture supernatant by sequential HiTrap chelating HP Ni^2+^-NTA and Superdex 75 columns (GE Healthcare). Before biolayer interferometry, purified CD81-LEL was stripped of its C-terminal His_6_ tag by digestion with carboxypeptidase A-agarose (Sigma).

Monoclonal antibody HCV1 was kindly provided by Yang Wang (MassBiologics, University of Massachusetts Medical School) (35). Monoclonal antibodies against different antigenic domains, CBH-4B and CBH-4G (domain A), HC-1 and HC-11 (domain B), CBH-7 (domain C), HC84.24 and HC84.26 (domain D), and HC33.1 and HC33.4 (domain E) were employed and prepared as described previously (6).

### Mass spectrometry.

Digestion was performed on 80 μg each of HEK293- and Sf9-derived sE2 by denaturing using 6 M guanidine HCl and 1 mM EDTA in 0.1 M Tris (pH 7.8), reduced with a final concentration of 20 mM dithiothreitol (DTT) (65°C for 90 min), and alkylated at a final concentration of 50 mM iodoacetamide (room temperature for 30 min). Samples were then buffer exchanged into 1 M urea in 0.1 M Tris (pH 7.8) for digestion. Sequential digestion was performed using trypsin (1/50 [wt/wt] enzyme/protein ratio) for 18 h at 37°C, followed by chymotrypsin (1/20 [wt/wt] enzyme/protein ratio) with 10 mM CaCl_2_ overnight at room temperature. LC-UV-MS analyses were performed using an UltiMate 3000 LC system coupled to an LTQ Orbitrap Elite mass spectrometer equipped with a heated electrospray ionization (HESI) source and operated in a top 5 dynamic exclusion mode. A volume of 30 μl (representing 9 μg of digested protein) of sample was loaded via the autosampler onto a C_18_ peptide column (AdvanceBio peptide 2.7 μm, 2.1 by 150 mm, part no. 653750-902; Agilent) enclosed in a thermostatted column oven set to 50°C. Samples were held at 6°C while queued for injection. The chromatographic gradient was conducted as described in Table 3. Glycan and glycopeptide identification was performed using the Byonic software and extracted ion chromatograms used for quantification of glycoforms in the Byologic software (Protein Metrics).

**TABLE 3:** Analytical gradient at 0.25 ml/min^*a*^

Time (min)	Flow (ml/min)	% A (0.1% FA in water)	% B (0.1% FA in ACN)	Divert valve	MS data collection
0.0	0.25	99	1	Waste	No
2.0	0.25	99	1	MS	Yes
6.0	0.25	90	10	MS	Yes
70.0	0.25	65	35	Waste	No
72.0	0.25	10	90	Waste	No
77.0	0.25	10	90	Waste	No
79.0	0.25	99	1	Waste	No
81.0	0.25	99	1	Waste	No
83.5	0.25	90	10	Waste	No
91.5	0.25	55	45	Waste	No
93.0	0.25	10	90	Waste	No
99.0	0.25	10	90	Waste	No
101.0	0.25	99	1	Waste	No
115.0	0.25	99	1	Waste	No

a FA, formic acid; ACN, acetonitrile.

### Glycoprotein modeling.

Glycans were modeled on the E2 protein structure using the Rosetta software (51). Prior to glycan modeling, residues from antigenic domain E were modeled into the structure of E2 core (PDB identifier [ID] 4MWF) (41) and refined using the FloppyTail method in Rosetta (52). The most abundant glycan at each site was encoded as an IUPAC string and modeled on E2 core using the SimpleGlycosylateMover in Rosetta, followed by the glycan_relax protocol to alleviate clashes involving glycans and energetically unfavorable conformations (using command line flags “-tree_based_min_pack -glycan_relax_refine”). Accessible surface area calculations were performed in Rosetta using the TotalSasa filter, specifying the probe radius using the “-sasa_calculator_probe_radius” command line flag. Percent E2 surface masking by glycans was calculated by dividing the surface accessibility of protein residues in the glycosylated E2 model by the surface accessibility of protein residues in the same model with all glycans removed. Structural visualization was performed in PyMOL (www.pymol.org).

### Biolayer interferometry.

The interaction of sE2 derived from HEK293 or Sf9 cells with CD81 and human monoclonal antibodies (HMAbs) in IgG format specific for antigenic domains A (CBH-4B and CBH-4G), B (HC-1 and HC-11), D (HC84.26 and HC84.24), and E (HCV1 and HC33.1) (6, 35) was measured using an Octet RED96 instrument and Ni^2+^-NTA biosensors (Pall FortéBio). The biosensors were loaded with 5 μg/ml purified His_6_-tagged sE2 for 600 s and then stabilized with the chemical cross-linker mixture of 0.1 M 1-ethyl-3-(3-dimethylaminopropyl)-carbodiimide and 0.025 M *N*-hydroxysuccinimide (GE Life Sciences) for 60 s, followed by reaction quenching with 1 M ethanolamine (pH 8.0) for 60 s. Association for 300 s followed by dissociation for 300 s against a 2-fold concentration dilution series of each antibody was performed. Data analysis was performed using Octet Data Analysis 10.0 software and utilized reference subtraction at 0 nM antibody concentration, alignment to the baseline, interstep correction to the dissociation step, and Savitzky-Golay fitting. Curves were globally fitted based on association and dissociation to obtain *K_D_* values.

### Animal immunization.

CD-1 mice were purchased from Charles River. Prior to immunization, sE2 antigens were formulated with SAS (Sigma adjuvant system) adjuvant (Sigma-Aldrich) at a 1:1 ratio according to the manufacturer’s instructions. On scheduled vaccination days, groups of 6 female mice, age 7 to 9 weeks, were injected via the intraperitoneal (i.p.) route with a 50 μg sE2 prime (day 0) and boosted with 10 μg sE2 on days 7, 14, 28, and 35. Blood samples were collected prior to each injection with a terminal bleed on day 49. The collected samples were processed for serum by centrifugation and stored at −80°C until analysis was performed.

### Competitive binding ELISA.

An ELISA measured the inhibition by mouse sera of HMAb binding to E2 glycoproteins captured by Galanthus nivalis lectin (GNA) (53). Briefly, microtiter plates were coated with GNA and blocked with 2.5% bovine serum albumin (BSA) and 2.5% normal goat serum in 0.1% phosphate-buffered saline (PBS) with Tween 20, prior to the addition of 2 μg/well HEK293 sE2. Pretitrated mouse serum was added to each well at a saturating concentration. After 1 h, HMAb was added at a concentration corresponding to 65% to 75% of the maximal optical density (OD) level, incubated for 30 min at either room temperature or 40°C, and then washed. Bound HMAb was detected by incubation with alkaline phosphatase-conjugated goat anti-human IgG (Promega), followed by incubation with *p*-nitrophenyl phosphate (Sigma) for color development. Absorbance was measured at 405/570 nm. This assay was carried out in triplicate, a minimum of two times for each HMAb.

### Cell lines and pseudoparticle plasmid construction.

The human embryonic kidney 293T (HEK293T) and Hep3B cells were purchased from the ATCC (www.atcc.org). Both HEK293T and Hep3B cells were grown in Dulbecco’s modified essential medium (DMEM; Thermo Fisher Scientific) supplemented with 10% fetal bovine serum (FBS) and 1% penicillin-streptomycin (Thermo Fisher Scientific). The human hepatoma cell line HuH7 was purchased from the European Collection of Authenticated Cell Cultures (ECACC) and routinely tested for mycoplasma contamination. HuH7 cells were grown in DMEM (Thermo Fisher Scientific) supplemented with 10% FBS and 1% nonessential amino acids (NEAA) (Thermo Fisher Scientific). All were maintained in a humidified 37°C, 5% CO_2_ incubator. The different genotype constructs for gt1a (H77c, UNKP1.1.1), gt1b (UNKP1.20.3, UNKP1.21.2), gt2a (J6, JFH), gt2b (UNKP2.4.1), gt2i (UNKP2.1.2), gt3 (UNKP3.2.2), gt4 (UNKP4.2.1), gt5 (UNKP5.1.1), and gt6 (UNKP6.1.1) were described previously (36, 54). The murine leukemia virus (MLV) Gag-Pol packaging vector (phCMV-5349) and luciferase reporter plasmid (pTG126) have been reported previously (36). All plasmids were grown and purified using the EndoFree plasmid maxi kit (Omega Bio-tek). Concentration and purity were verified by restriction analysis, sequencing, and spectrophotometry.

### Pseudoparticle generation and titration.

HCV pseudoparticles (HCVpps) were generated by cotransfection of HEK293T cells with the MLV Gag-Pol packaging vector, luciferase reporter plasmid, and plasmid expressing HCVE1E2 using Lipofectamine 3000 (Thermo Fisher Scientific), as described previously (55). A no-envelope control (empty plasmid) was used as negative control in all experiments. Supernatants containing HCVE1E2 pseudoparticles were harvested at 48 h and 72 h posttransfection and filtered through 0.45-μm-pore-size membranes. The generated HCVpp is capable of achieving a single-round infection in Hep3B or HuH7 target cells and contains a luciferase reporter gene that can be expressed in infected cells. Titration of HCVpp was then determined by infecting Hep3B cells with a serial dilution.

### HCVpp neutralization assays.

For infectivity and neutralization testing of HCVpp, either 10^4^ Hep3B cells per well or 1.5 × 10^4^ HuH7 cells per well were plated in white 96-well tissue culture plates (Corning) and incubated overnight at 37°C. The following day, HCVpps were mixed with appropriate amounts of antibody and then incubated for 1 h at 37°C before adding to Hep3B or HuH7 cells. After 72 h at 37°C, either 100 μl Bright-Glo (Promega) was added to each well and incubated for 2 min or cells were lysed with cell lysis buffer (catalog no. E1500; Promega) and placed on a rocker for 15 min. Luciferase activity was then measured in relative light units (RLUs) using either a SpectraMax M3 microplate reader (Molecular Devices) with the SoftMax Pro6 software (Bright-Glo protocol), or wells were individually injected with 50 μl luciferase substrate and read using a FLUOstar Omega plate reader (BMG Labtech) with the MARS software. Infection by HCVpp was measured in the presence of anti-HCV E2 MAbs, tested animal sera, preimmune animal sera, and nonspecific IgG at the same dilution. Each sample was tested in duplicate or triplicate. Neutralizing activities were reported as 50% inhibitory dilution (ID_50_) values and were calculated by nonlinear regression (GraphPad Prism version 7), using lower and upper bounds (0% and 100% inhibition) as constraints to assist curve fitting.

### Statistical analysis.

Comparison of neutralization titers between groups (ID_50_ values; Fig. 8 and 9) was performed using Kruskal-Wallis analysis of variance (ANOVA) with Dunn’s multiple-comparison test. Data analysis was not blinded. Differences were considered statistically significant at a *P* value of <0.05. Comparison of serum antibody percent E2 binding competition between groups (Fig. 7) was performed using a *t* test. Statistical analyses were performed using the GraphPad Prism 7 software.

## Supplementary Material

Supplemental file 1
